# SENESCENCE-DERIVED PROTEIN BIOMARKERS DURING AGING AND OSTEOARTHRITIS

**DOI:** 10.1093/geroni/igac059.782

**Published:** 2022-12-20

**Authors:** Birgit Schilling, Sandip Patel, Jacob Rose, Judith Campisi, Joanna Bons, Christina King, Charles Schurman

**Affiliations:** Buck Institute for Research on Aging, Novato, California, United States; Buck Institute, Novato, California, United States; Buck Institute, Novato, California, United States; Buck Institute, Novato, California, United States; Buck Institute, Novato, California, United States; Buck Institute, Novato, California, United States; Buck Institute, Novato, California, United States

## Abstract

Aging is a complex biological process associated with progressive loss of physiological function and susceptibility to several diseases, such as cancer and neurodegeneration. As senescence burden increases with aging and becomes a risk factor for many age-related diseases we are specifically interested in senescence-derived aging signatures. We use cutting-edge proteomic workflows to investigate both the senescence-associated secretory phenotype (SASP) in tissue cultures. We are subsequently examining exosome proteins as well as lipid cargo in plasma from human young (20–26 yrs) and old cohorts and old (60–66 yrs) individuals. We will also present current work assessing senescence markers in cartilage and bone targeting underlying mechanisms and options for intervention for osteoarthritis. Overall, our focus is specifically directed towards senescence-derived biomarkers of aging.

